# Triglyceride-glucose index demonstrates false-positive association with cardiometabolic multimorbidity progression in cardiometabolic disease patients: Observation and Mendelian randomization study

**DOI:** 10.1097/MD.0000000000047632

**Published:** 2026-02-13

**Authors:** Zekai Zhou, Heng Wee Tan, Yubo Zhang, Le Yu, Yanping Wang, Songming Chen, Chujuan Zeng

**Affiliations:** a Department of Cardiology, The First Affiliated Hospital of Shantou University Medical College, Shantou, Guangdong, People’s Republic of China; b The Second Affiliated Hospital of Shantou University Medical College, Shantou, Guangdong, People’s Republic of China; c Laboratory of Cancer Biology and Epigenetics, Department of Cell Biology and Genetics, Shantou University Medical College, Shantou, Guangdong, People’s Republic of China.

**Keywords:** cardiometabolic multimorbidity, Cohort study, insulin resistance, Mendelian randomization, Triglyceride Glucose index

## Abstract

The Triglyceride-Glucose (TyG) index is a recognized predictor for incident cardiometabolic diseases (CMDs) and cardiometabolic multimorbidity (CMM) in general populations. However, its utility for predicting progression from single CMD to CMM among patients with existing CMD remains unverified. This study combined retrospective cohort analyses (cross-sectional cohorts: China Health and Retirement Longitudinal Study [CHARLS]-1, n = 5415; First Affiliated Hospital of Shantou University Medical Center, n = 544; longitudinal cohort: CHARLS-2, n = 1866) with Mendelian randomization to evaluate this relationship. Cross-sectional analyses initially indicated positive associations between elevated TyG index and CMM progression (per-standard deviation increase: CHARLS-1 adjusted odds ratio [OR] = 1.43, 95% confidence interval [CI]: 1.30–1.57; First Affiliated Hospital of Shantou University Medical Center adjusted OR = 1.75, 95% CI: 1.33–2.31). However, longitudinal analysis showed no significant association after multivariable adjustment (hazard ratio = 0.87, 95% CI: 0.73–1.02). Crucially, Mendelian randomization analysis with sequential exclusion of confounder-associated single nucleotide polymorphisms (glucose, triglycerides, and body mass index) revealed no causal relationship (Model 3 inverse-variance weighted OR = 0.647, 95% CI: 0.412–1.014). These findings demonstrate that the apparent association between TyG index and CMM progression in patients with baseline CMD is likely a false-positive result attributable to residual confounding, with no causal link supported by rigorous longitudinal or genetic evidence. Thus, while TyG is valuable for predicting initial CMD onset, it lacks clinical utility for forecasting progression to multimorbidity in established patients, necessitating exploration of alternative biomarkers for this critical transition.

## 1. Introduction

Multimorbidity refers to the presence of at least 2 chronic conditions in a person, and it has seen a marked rise in global prevalence in recent years, driven mainly by rapid population aging. Current estimates indicate that nearly one-third of the worldwide population, including in China, is affected by multimorbidity, with prevalence rates increasing sharply in the older age groups.^[1,2]^ Of particular concern is cardiometabolic multimorbidity (CMM) – the coexistence of 2 or more cardiometabolic diseases (CMDs), such as diabetes, stroke, and coronary heart disease (CHD) which poses a substantial public health and clinical challenge.^[3]^ Notably, CMM is associated with significantly higher mortality rates compared to a single CMD, with this disparity being particularly pronounced in Chinese populations.^[3,4]^

Substantial evidence has established insulin resistance as a key pathophysiological mechanism underlying various CMDs, including diabetes mellitus, stroke, and CHD.^[5,6]^ The Triglyceride-Glucose (TyG) index, calculated as Ln (fasting triglycerides [mg/dL] × fasting glucose [mg/dL]/2), has emerged as a reliable surrogate marker for insulin resistance, demonstrating high diagnostic accuracy across multiple validation studies.^[7]^ The TyG index has been extensively associated with various cardiometabolic disorders. Specifically, it demonstrates a dose-dependent relationship with diabetes incidence.^[8]^ Furthermore, elevated TyG index levels significantly correlate with diabetic microvascular and macrovascular complications.^[9]^ The TyG index also holds significant clinical implications for cardiovascular diseases and stroke caused by cardiometabolic risk associated with diabetes. For stroke, the TyG index is significantly associated with both stroke incidence and stroke-related mortality.^[10]^ For cardiovascular diseases, TyG index not only serves as a biomarker for cardiovascular diseases detection but also independently predicts cardiovascular risk.^[11]^ Cumulatively, these findings highlight the TyG index’s dual role as both a diagnostic tool and a prognostic predictor in CMD management.

Over the past few years, several predictive indicators for CMM have been developed, and these include TyG,^[12,13]^ body mass index (BMI),^[14]^ Chinese visceral adiposity index,^[12]^ visceral adiposity index,^[15]^ body roundness index,^[16]^ and indicators associated with insulin resistance in obesity.^[12,17]^ However, CMM development typically follows a progressive trajectory, initiating with a single CMD before advancing to multimorbidity. To date, no systematic studies have investigated predictive indicators for this transitional process: from single CMD to CMM.

Despite the well-documented predictive value of the TyG index for individual CMDs and CMM, its specific predictive value for CMM progression in patients with preexisting CMDs remains underexplored. To address this knowledge gap, we systematically evaluated the TyG index’s predictive capacity for CMM development in 2 Chinese CMD patient populations: China Health and Retirement Longitudinal Study (CHARLS) and First Affiliated Hospital of Shantou University Medical Center (FAHSUMC). Specifically, a cohort from the FAHSUMC representing region-specific data from southern China and 2 nationally representative cohorts from the CHARLS (based on cross-sectional and longitudinal analyses) were used in the current study. This multi-cohort approach enabled the investigation of the TyG index’s ability to predict the onset of CMM in patients with baseline CMD diagnoses.

## 2. Methods

### 2.1. Study design

**Figure 1.**
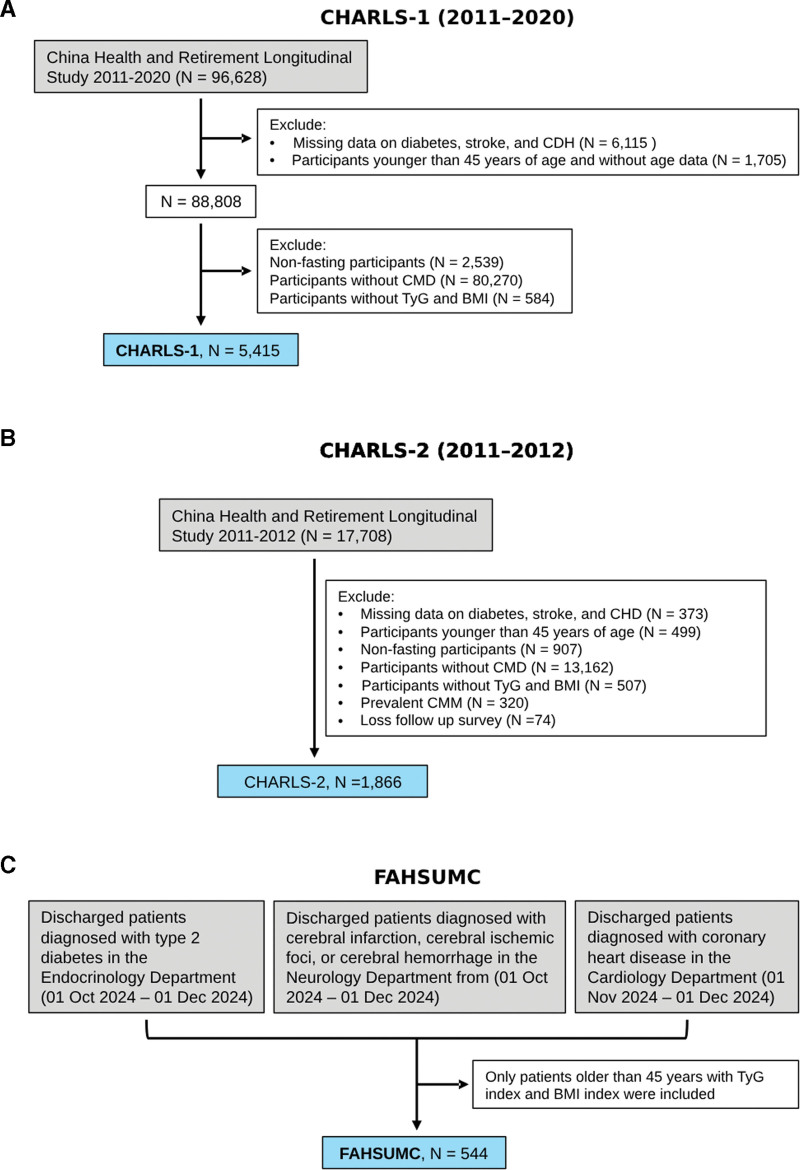
Flowchart of participant selection in the 3 cohorts of this study: (A) China Health and Retirement Longitudinal Study (CHARLS)-1 (2011–2020) for cross-sectional analysis, (B) CHARLS-2 (2011–2012) for longitudinal analysis, and (C) First Affiliated Hospital of Shantou University Medical Center (FAHSUMC) for cross-sectional validation.

This study analyzed data from 2 independent population studies: CHARLS and FAHSUMC. The CHARLS study was approved by Peking University’s Biomedical Ethics Committee (IRB00001052-11015) and conducted following the Declaration of Helsinki.^[18]^ The FAHSUMC study received approval from its respective institutional review board (No. B-2025-021, The First Affiliated Hospital of Shantou University). We implemented a hybrid design combining cross-sectional and longitudinal analyses to examine TyG-CMM associations in CMD patients (Fig. 1C). The CHARLS cohort was stratified into 2 analytical subsets: CHARLS-1 (cross-sectional analysis, Fig. 1A) and CHARLS-2 (longitudinal analysis, Fig. 1B), using distinct inclusion criteria. To reduce recall bias, the outcome of CMM in CHARLS-2 was defined as the first occurrence of CMM during follow-up visits between 2013 and 2020. For participants not completing full follow-up and without CMM, their status was determined based on the last available follow-up data. The median follow-up time was 9 years. The FAHSUMC data was predominantly utilized for cross-sectional validation. Finally, Mendelian randomization (MR) analysis was conducted to validate causal inferences. The schematic overview of the data extraction workflow was summarized in Figure 1.

### 2.2. Data collection and definitions

The CHARLS database provided baseline covariates including sex, age, smoking status, alcohol use, recent healthcare utilization (hospitalizations within 1 year and outpatient visits within 1 month), household income, hypertension status, systolic blood pressure (SBP)/diastolic blood pressure, anthropometric measures (waist circumference and BMI), sleep duration, metabolic biomarkers (fasting plasma glucose [FPG], total cholesterol [TC], triglycerides [TG], high-density lipoprotein, low-density lipoprotein, glycated hemoglobin [HbA1c], uric acid, C-reactive protein [CRP], and leisure-time physical activity).

Diabetes was defined according to the criteria of the “Clinical Guidelines for Prevention and Treatment of Type 2 Diabetes Mellitus in the Elderly in China (2022 Edition)”: HbA1c level ≥ 6.5% or FPG ≥ 7 mmol/L, random blood glucose level ≥ 11.1 mmol/L,^[19]^ or based on self-reported physician diagnosis (*Have you ever been diagnosed with diabetes or high blood sugar by a doctor?*). The determination of whether participants have CHD or stroke is primarily based on self-reported data collected during baseline and follow-up surveys (*Have you ever been diagnosed with heart disease, CHD, angina pectoris, congestive heart failure, or other heart problems by a doctor?* and *Have you ever been diagnosed with stroke by a doctor?*). Smoking and alcohol use were dichotomized based on lifetime exposure history (*ever smoked* and *ever consumed alcohol*). Leisure-time physical activity encompassed structured exercises, including dance, gym workouts, and “qigong” practice.

The FAHSUMC cohort provided comparable baseline variables, including demographic characteristics (age and sex), lifestyle factors (smoking and alcohol use), clinical parameters (hypertension status and SBP/diastolic blood pressure), anthropometrics (height, weight, and BMI), and metabolic biomarkers (FPG, TC, TG, high-density lipoprotein, low-density lipoprotein, HbA1c, CRP, and uric acid). Clinical diagnoses of type 2 diabetes, CHD, and hypertension were extracted from electronic medical records. Stroke diagnoses encompassed cerebral infarction, transient ischemic attack, and intracerebral hemorrhage. Smoking/alcohol use histories were abstracted from medical records. Laboratory parameters were obtained from the most recent clinical assessments. The detailed inclusion criteria for data are presented in Figure 1. In addition to the primary analytical data (diabetes, stroke, CHD, TyG index, and BMI), we choose to ignore missing values when processing data. Details of the missing values are listed in Tables S4 and S5, Supplemental Digital Content, https://links.lww.com/MD/R393.

The TyG indexes are calculated as follows: TyG = Ln (fasting TG [mg/dL] × fasting glucose [mg/dL]/2). Follow-up duration was calculated as the last follow-up date minus the initial enrollment date, expressed in years.

### 2.3. Two-sample MR analysis

Single nucleotide polymorphism (SNP) data were obtained from published genome-wide association studies (GWAS). TyG index-associated SNPs were sourced from Si et al and directly adopted from the original study, which is a study explore the relationship between TyG and cardio-cerebrovascular diseases.^[20]^ This study derived from the UK Biobank cohort by excluding individuals with diabetes or lipid metabolism disorders (n = 273,368) to minimize medication confounding. For TyG index analysis, GWAS testing identified 212 SNPs meeting quality control thresholds (minor allele frequency > 0.01, missing rate < 0.05, Hardy–Weinberg *P* < 1 × 10^−5^, genome-wide *P* < 5 × 10^−8^). These SNPs underwent refinement via LD clumping (*R*^2^ < 0.01, clustering window ≥ 1000 kb) and yielding 192 final instrumental SNPs (Table S8, Supplemental Digital Content, https://links.lww.com/MD/R393).

These SNPs were stratified into 3 models with sequential adjustment for confounders: Model 1 retained 183 SNPs after MR-PRESSO outlier removal without excluding any confounders; Model 2 excluded 119 SNPs strongly associated with FPG and TG (FPG and TG’s SNPs sourced also from Si et al), resulting in 67 SNPs post-MR-PRESSO outlier removal (remove 6 SNPs); and Model 3 further excluded 3 additional SNPs associated with BMI and metabolic syndrome (SNP data obtained from https://www.ebi.ac.uk/gwas/), and we got 66 SNPs after MR-PRESSO outlier removal (remove 4 SNPs). We ensured all exhibiting F-statistics (β^2^/SE^2^) above the threshold of 10 among 3 model, indicating strong instrument strength and minimal risk of weak instrument bias. SNPs associated with CMM were derived from a published GWAS by Zhao C et al (GCST90274723) also the population from the UK Biobank.^[21]^

The 3 core IV assumptions were verified: relevance (minor allele frequency > 0.01, genome-wide *P* < 5 × 10^−8^), independence (3 adjusted model), and exclusion restriction (MR-Egger intercept *P* > .05).

Primary MR analyses used inverse-variance weighted (IVW) regression, which provides robust estimates under balanced pleiotropy.^[22]^ Heterogeneity was quantified using Cochran Q statistic (*P* < .05 indicates significant heterogeneity). Random-effects IVW models were applied when heterogeneity was detected; otherwise, fixed-effects models were used. MR-Egger intercept tests evaluated directional pleiotropy (significant when intercept ≠ 0, *P* < .05). MR Funnel and leave-one-out sensitivity analysis also use for sensitive analyze. Both MR Egger and weighted median methods were further utilized to corroborate the findings.

### 2.4. Statistical analysis

Normality distribution was assessed using Kolmogorov–Smirnov tests for CHARLS-1 (N > 5000) and Shapiro–Wilk tests for FAHSUMC, CHARLS-2 cohorts (N < 5000) based on sample size thresholds recommended by the central limit theorem. Continuous variables are reported as mean ± standard error or median (interquartile range), with categorical variables expressed as frequencies (%). Group comparisons employed analysis of variance for continuous variables and χ^2^ tests for categorical variables.

In both cross-sectional and longitudinal analyses, multivariable regression models were employed to assess TyG-associated CMM risk. For cross-sectional data, logistic regression generated adjusted odds ratios (ORs) with 95% confidence intervals (CIs) across TyG tertiles; for longitudinal data, Cox proportional hazards models yielded adjusted hazard ratios with 95% CIs. Three sequential adjustment models (Model 1–3) were constructed in both phases. TyG was further analyzed as a continuous exposure, with effect estimates expressed per 1-standard deviation increment to quantify dose–response relationships. Multivariable restricted cubic splines (3 knots) evaluated potential nonlinear associations between TyG and CMM outcomes.

Our subgroup analysis focuses on various cardiometabolic risks^[23]^ and demographic characteristics. We conducted subgroup analyses based on gender (male and female), age (≤ 65 and > 65 years), smoking status (yes/no), alcohol consumption (yes/no), hypertension (yes/no), hypercholesterolemia (≤ 297.44 mg/dL and > 297.44 mg/dL), hyperuricemia (≤ 7.20 mg/dL and > 7.20 mg/dL), high CRP levels (≤ 8 mg/dL and > 8 mg/dL), and overweight status (BMI ≤ 24 and > 24). All analyses were conducted using R version 4.4.1 (https://www.r-project.org/), and a *P*-value < .05 was considered statistically significant.

## 3. Results

### 3.1. Baseline CMD-related characteristics of study participants

Pooled analysis of cross-sectional data from CHARLS-1 (Table S1, Supplemental Digital Content, https://links.lww.com/MD/R393) and FAHSUMC (Table S2, Supplemental Digital Content, https://links.lww.com/MD/R393) revealed significant progressive elevations in hypertension prevalence, CMM incidence, alcohol consumption prevalence, SBP, TC, HbA1c, and uric acid levels with ascending TyG index tertiles. Moreover, longitudinal data from the CHARLS-2 cohort demonstrated concordant findings (Table S3, Supplemental Digital Content, https://links.lww.com/MD/R393). Detailed normality test outcomes for continuous variables are provided in Tables S6 and S7, Supplemental Digital Content, https://links.lww.com/MD/R393.

### 3.2. Cross-sectional analyze the associations between the TyG index and the risk of CMM in CMD patients

**Figure 2.**
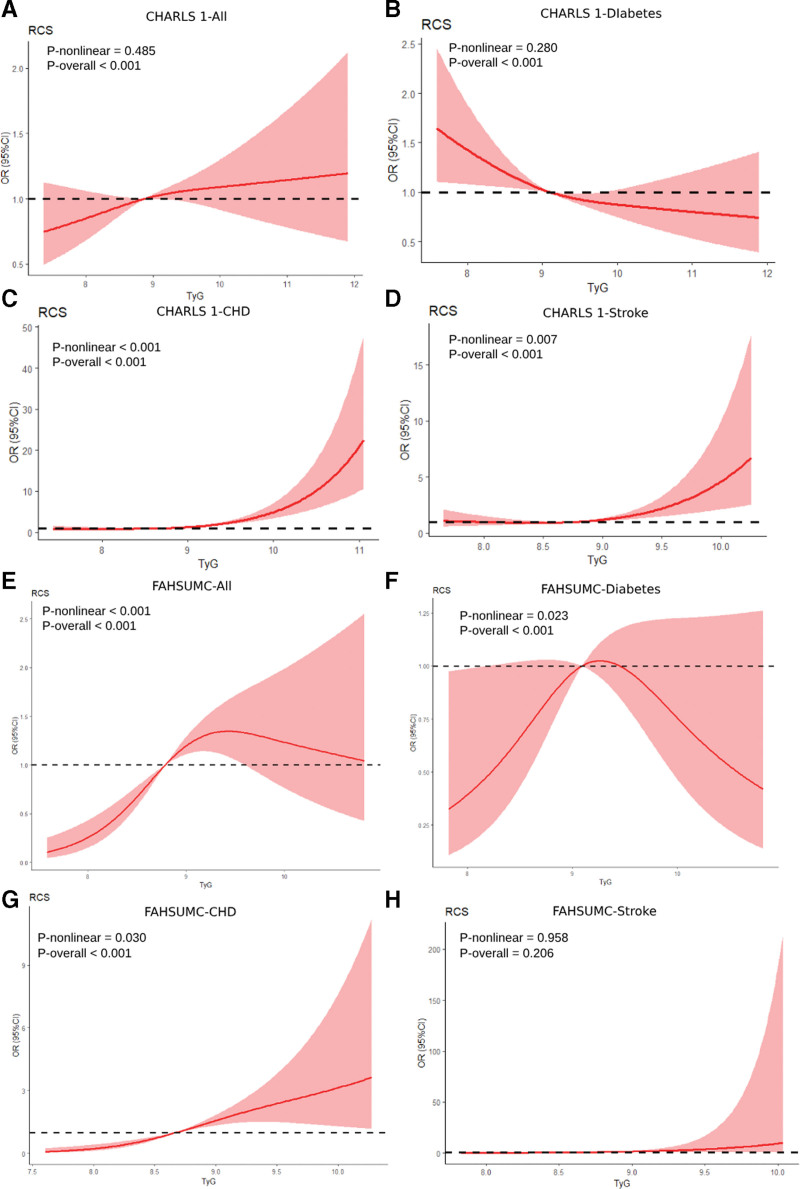
Investigation of CMD-to-CMM transition among 2 cross-sectional design cohorts. CHARLS-1: all participants (A), only with baseline diabetes (B), only with baseline CHD (C) and only with baseline stroke (D). Adjusted for age, sex, hypertension, smoking, alcohol use, HDL, LDL, BMI, SBP, DBP, CRP, urea acid and HbA1c; FAHSUMC: all participants (E), only with baseline diabetes (F), only with baseline CHD (G) and only with baseline stroke (H). Adjusted for age, sex, hypertension, smoking, alcohol use, HDL, LDL, BMI, CRP and urea acid. BMI = body mass index, CHARLS = China Health and Retirement Longitudinal Study, CHD = coronary heart disease, CMD = cardiometabolic diseases, CMM = cardiometabolic multimorbidity, CRP = C-reactive protein, DBP = diastolic blood pressure, FAHSUMC = First Affiliated Hospital of Shantou University Medical Center, HDL = high-density lipoprotein, LDL = low-density lipoprotein, SBP = systolic blood pressure.

To assess TyG’s predictive utility for CMM in CMD patients, we analyzed cross-sectional cohorts CHARLS-1 and FAHSUMC. Elevated TyG associated with increased CMM risk in both cohorts. Per-standard deviation TyG increase yielded adjusted ORs of 1.43 (95% CI: 1.30–1.57) in CHARLS-1 and 1.75 (95% CI: 1.33–2.31) in FAHSUMC. Categorically (Q3 vs Q1), risks rose to OR = 1.36 (95% CI: 1.09–1.68) and OR = 3.95 (95% CI: 2.13–7.34), respectively (Table 1). Restricted cubic splines (RCS) demonstrated linear (CHARLS-1) and nonlinear (FAHSUMC) TyG-CMM risk relationships (Fig. 2A, E). The RCS plots for different CMD subgroups in the CHARLS-1 cohort are shown in Figure 2B–D, while those for the FAHSUMC cohort are displayed in Figure 2F–H.

**Table 1:** Associations between the TyG index and the risk of CMM in CMD patients (cross-sectional).

	Q1	Q2	*P* value	Q3	*P* value	Per-SD increase	*P* value
CHLARS-1							
All participants (n = 5415)							
Model 1	Ref.	1.34 (1.11–1.61)	.002	1.99 (1.67–2.38)	<.001	1.41 (1.29–1.55)	<.001
Model 2	Ref.	1.24 (1.02–1.51)	.035	1.86 (1.52–2.28)	<.001	1.41 (1.26–1.58)	<.001
Model 3	Ref.	1.11 (0.91–1.35)	.321	1.36 (1.09–1.68)	.006	1.43 (1.30–1.57)	<.001
Only baseline with diabetes (n = 3150)							
Model 1	Ref.	0.93 (0.77–1.13)	.491	0.92 (0.75–1.11)	.374	0.94 (0.85–1.04)	.246
Model 2	Ref.	0.82 (0.66–1.01)	.059	0.79 (0.63–0.99)	.039	0.86 (0.76–0.98)	.023
Model 3	Ref.	0.78 (0.63–0.97)	.026	0.74 (0.58–0.94)	.014	0.81 (0.71–0.94)	.004
Only baseline with CHD (n = 2722)							
Model 1	Ref.	1.52 (1.21–1.92)	<.001	4.48 (3.61–5.55)	<.001	3.33 (2.88–3.84)	<.001
Model 2	Ref.	1.51 (1.18–1.93)	.001	4.37 (3.40–5.62)	<.001	3.59 (3.02–4.25)	<.001
Model 3	Ref.	1.30 (0.99–1.71)	.063	2.54 (1.90–3.40)	<.001	2.33 (1.90–2.85)	<.001
Only baseline with stroke (n = 535)							
Model 1	Ref.	1.22 (0.80–1.86)	.365	4.06 (2.61–6.33)	<.001	2.70 (2.02–3.62)	<.001
Model 2	Ref.	1.22 (0.77–1.94)	.396	3.62 (2.17–6.04)	<.001	2.60 (1.85–3.64)	<.001
Model 3	Ref.	1.10 (0.66–1.83)	.706	2.32 (1.29–4.16)	.005	1.83 (1.24–2.70)	.002
FAHSUMC							
All participants (n = 544)							
Model 1	Ref.	2.33 (1.49–3.62)	<.001	3.17 (2.04–4.94)	<.001	1.67 (1.33–2.09)	<.001
Model 2	Ref.	2.73 (1.71–4.36)	<.001	3.70 (2.28–5.9)	<.001	1.89 (1.47–2.41)	<.001
Model 3	Ref.	3.31 (1.83–5)	<.001	3.95 (2.13–7.34)	<.001	1.75 (1.33–2.31)	<.001
Only baseline with diabetes (n = 294)							
Model 1	Ref.	0.65 (0.35–1.20)	.166	0.62 (0.34–1.14)	.126	0.69 (0.51–0.94)	.017
Model 2	Ref.	0.91 (0.46–1.80)	.783	0.92 (0.46–1.84)	.815	0.85 (0.60–1.20)	.354
Model 3	Ref.	1.24 (0.54–2.85)	.607	1.35 (0.55–3.30)	.508	0.99 (0.64–1.54)	.969
Only baseline with CHD (n = 331)							
Model 1	Ref.	3.27 (1.86–5.76)	<.001	5.81 (3.25–10.39)	<.001	3.17 (2.21–4.55)	<.001
Model 2	Ref.	3.37 (1.86–6.10)	<.001	5.52 (2.92–10.42)	<.001	3.45 (2.30–5.17)	<.001
Model 3	Ref.	4.30 (2.07–8.95)	<.001	6.76 (3.04–15.04)	<.001	4.15 (2.49–6.90)	<.001
Only baseline with stroke (n = 164)							
Model 1	Ref.	2.78 (1.28–6.07)	.01	13.91 (4.80–40.31)	<.001	5.50 (2.76–10.95)	<.001
Model 2	Ref.	2.83 (1.21–6.60)	.016	16.80 (5.36–52.64)	<.001	5.66 (2.72–11.74)	<.001
Model 3	Ref.	2.68 (0.92–7.82)	.071	5.94 (1.78–19.88)	.004	3.87 (1.61–9.33)	.002

Covariate for CHARLS1. Model 1: Unadjusted. Model 2: Adjusted for age, sex, hypertension, smoking, alcohol use, HDL, and LDL. Model 3: Adjusted for age, sex, hypertension, smoking, alcohol use, HDL, LDL, BMI, SBP, DBP, CRP, urea acid, and HbA1c.

Covariate for FAHSUMC. Model 1: Unadjusted. Model 2: Adjusted for age, sex, hypertension, smoking, and alcohol use. Model 3: Adjusted for age, sex, hypertension, smoking, alcohol use, HDL, LDL, BMI, CRP, and urea acid.

BMI = body mass index, CHARLS = China Health and Retirement Longitudinal Study, CHD = coronary heart disease, CMD = cardiometabolic diseases, CMM = cardiometabolic multimorbidity, CRP = C-reactive protein, DBP = diastolic blood pressure, FAHSUMC = First Affiliated Hospital of Shantou University Medical Center, HbA1c = glycated hemoglobin, HDL = high-density lipoprotein, LDL = low-density lipoprotein, SBP = systolic blood pressure, SD = standard deviation, TyG = triglyceride-glucose.

Stratification by baseline CMD status revealed differential TyG effects. In subgroups with only baseline CHD or only baseline stroke, TyG consistently predicted CMM progression across both CHARLS-1 and FAHSUMC. By contrast, within the only baseline diabetes subgroup, TyG showed no significant association in FAHSUMC (OR = 0.99, 95% CI: 0.64–1.54) and an inverse association in CHARLS-1, where a protective effect was observed (OR = 0.81, 95% CI: 0.71–0.94; Table 1). This indicates TyG’s predictive capacity depends on baseline CMD phenotype.

### 3.3. longitudinal analyze the associations between the TyG index and the risk of CMM in CMD patients

**Figure 3.**
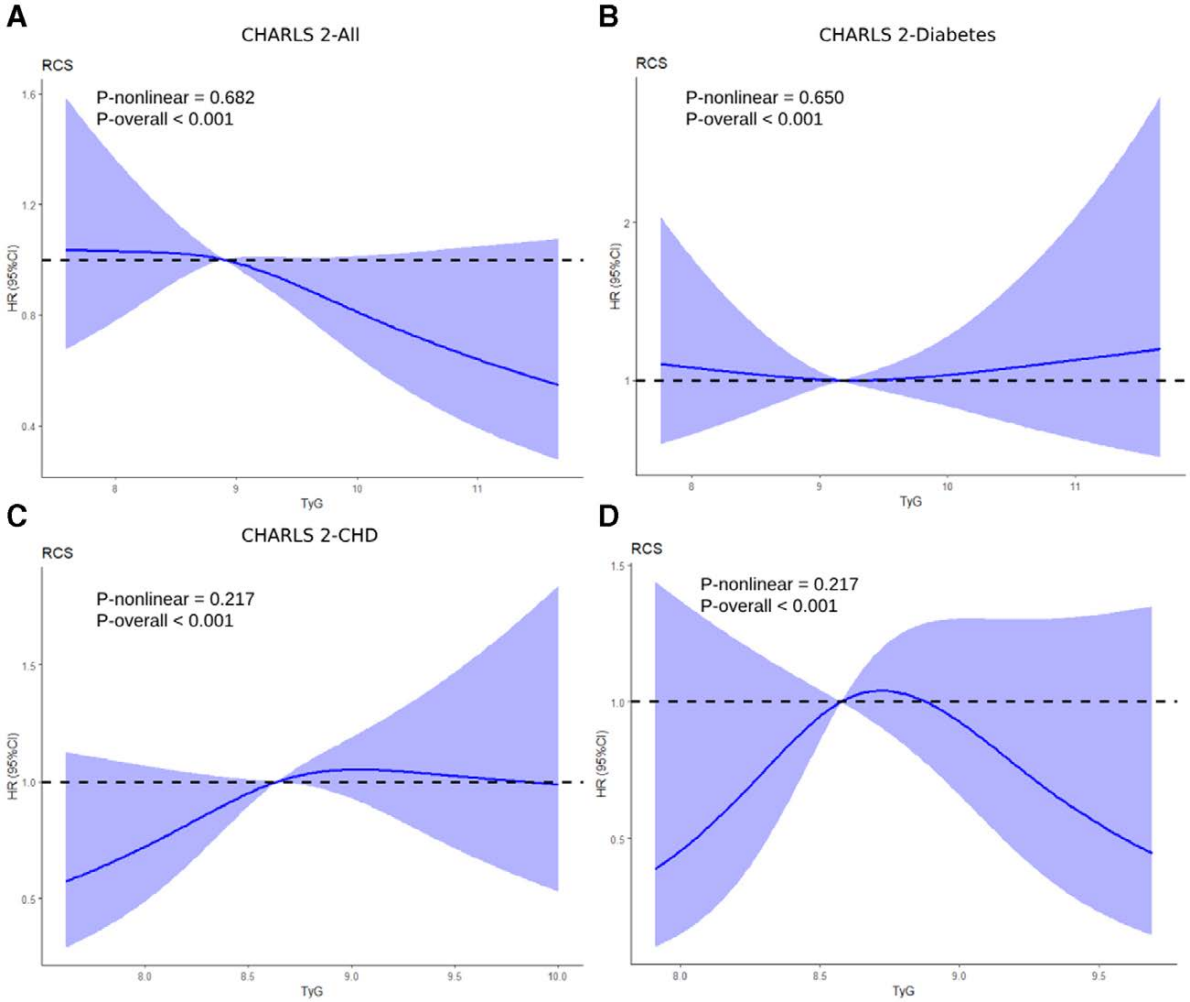
Investigation of CMD-to-CMM transition in longitudinal design cohort. All participants (A), only with baseline diabetes (B), only with baseline CHD (C) and only with baseline stroke (D). Adjusted for age, sex, hypertension, smoking, alcohol use, HDL, LDL, BMI, SBP, DBP, CRP, urea acid and HbA1c. BMI = body mass index, CHARLS = China Health and Retirement Longitudinal Study, CHD = coronary heart disease, CMD = cardiometabolic diseases, CMM = cardiometabolic multimorbidity, CRP = C-reactive protein, DBP = diastolic blood pressure, HDL = high-density lipoprotein, LDL = low-density lipoprotein, SBP = systolic blood pressure.

To further examine TyG-CMM associations in CMD, we utilized longitudinal cohort CHARLS-2. TyG correlated with CMM only in unadjusted Model 1 (Q3 vs Q1, hazard ratio = 1.31, 95% CI: 1.05–1.63), with significance attenuated upon covariate adjustment (Table 2). Multivariable RCS curves indicated a linear inverse TyG-CMM relationship (Fig. 3A). When stratified by baseline conditions (only baseline diabetes, only baseline CHD, and only baseline stroke), TyG-CMM associations persisted solely in the CHD subgroup (Models 1–2), disappearing after full adjustment (Model 3; Table 2). The multivariable RCS plots for different CMD subgroups in the CHARLS-1 cohort are shown in Figure 3B–D. Given TyG-CMM links were observable only in cross-sectional analyses and unadjusted longitudinal models, these findings suggest a likely false-positive relationship.

**Table 2:** Associations between the TyG index and the risk of CMM in CMD patients (longitudinal).

	Q1	Q2	*P* value	Q3	*P* value	Per-SD increase	*P* value
CHARLS 2							
ALL participants (n = 1866)							
Model 1	Ref.	1.15 (0.92–1.45)	.23	1.31 (1.05–1.63)	.016	1.09 (0.98–1.21)	.129
Model 2	Ref.	0.90 (0.71–1.15)	.411	1.02 (0.79–1.31)	.904	1.09 (0.97–1.22)	.129
Model 3	Ref.	0.88 (0.69–1.12)	.301	0.85 (0.65–1.13)	.264	0.87 (0.73–1.02)	.089
Only baseline with diabetes (n = 1033)							
Model 1	Ref.	1.00 (0.71–1.43)	.989	1.58 (1.15–2.18)	.005	1.18 (1.02–1.37)	.026
Model 2	Ref.	0.77 (0.54–1.12)	.173	1.28 (0.88–1.86)	.189	1.16 (0.99–1.36)	.062
Model 3	Ref.	0.73 (0.50–1.06)	.101	1.06 (0.71–1.58)	.781	1.02 (0.80–1.29)	.897
Only baseline with CHD (n = 720)							
Model 1	Ref.	1.57 (1.10–2.23)	.013	1.90 (1.35–2.67)	<.001	1.63 (1.28–2.07)	<.001
Model 2	Ref.	1.46 (1.00–2.14	.048	1.64 (1.09–2.47)	.018	1.64 (1.28–2.11)	<.001
Model 3	Ref.	1.37 (0.93–2.01)	.107	1.28 (0.84–1.96)	.256	1.21 (0.87–1.67)	.257
Only baseline with stroke (n = 113)							
Model 1	Ref.	3.29 (1.31–8.30)	.011	2.44 (0.95–6.28	.065	1.46 (0.89–2.39)	.134
Model 2	Ref.	1.86 (0.71–4.88)	.21	1.21 (0.42–3.44)	.726	1.35 (0.78–2.34)	.289
Model 3	Ref.	1.86 (0.66–5.21)	.239	0.97 (0.32–2.95)	.957	0.83 (0.41–1.70)	.617

Model 1: Unadjusted. Model 2: Adjusted for age, sex, hypertension, smoking, alcohol use, HDL, and LDL. Model 3: Adjusted for age, sex, hypertension, smoking, alcohol use, HDL, LDL, BMI, SBP, DBP, CRP, urea acid, and HbA1c.

BMI = body mass index, CHARLS = China Health and Retirement Longitudinal Study, CHD = coronary heart disease, CMD = cardiometabolic diseases, CMM = cardiometabolic multimorbidity, CRP = C-reactive protein, DBP = diastolic blood pressure, HbA1c = glycated hemoglobin, HDL = high-density lipoprotein, LDL = low-density lipoprotein, SBP = systolic blood pressure, SD = standard deviation, TyG = triglyceride-glucose.

### 3.4. MR analyze the relationship between TyG and CMM

To investigate the causal relationship between TyG and CMM, we performed MR analysis. Among the 3 models, a significant association between TyG and CMM was observed only in Model 1 without confounder exclusion (IVW OR = 0.607, 95% CI: 0.477–0.771; Table 3). However, SNPs in Model 1 exhibited horizontal pleiotropy (Egger intercept = 0.0144, *P* < .001; Table S9, Supplemental Digital Content, https://links.lww.com/MD/R393). In contrast, no association was found between TyG and CMM in Models 2 and 3 after confounder adjustment (Model 2: IVW OR = 0.772, 95% CI: 0.501–1.118; Model 3: IVW OR = 0.647, 95% CI: 0.412–1.014). Furthermore, neither Model 2 nor Model 3 showed evidence of horizontal pleiotropy (Model 2: Egger intercept = −0.012, *P* = .091; Model 3: Egger intercept = 0.001, *P* = .202). These results suggest no causal relationship between TyG and CMM. Other sensitivity analyses are detailed in Figures S1 to S3, Supplemental Digital Content, https://links.lww.com/MD/R393.

**Table 3:** Mendelian randomization results.

Exposure	Methods	B	SE	*P* value	OR	Lower	Upper
Model 1	IVW	−0.500	0.122	<.001	0.607	0.477	0.771
	MR Egger	0.070	0.191	.713	1.073	0.738	1.559
	Weighted median	−0.132	0.135	.330	0.877	0.672	1.143
	Simple mode	−0.486	0.428	.258	0.615	0.266	1.423
	Weighted mode	0.106	0.149	.478	1.112	0.830	1.490
Model 2	IVW	−0.259	0.220	.239	0.772	0.501	1.188
	MR Egger	0.138	0.316	.664	1.148	0.618	2.134
	Weighted median	−0.084	0.201	.678	0.920	0.620	1.365
	Simple mode	−0.532	0.469	.262	0.587	0.234	1.473
	Weighted mode	−0.128	0.154	.408	0.880	0.651	1.189
Model 3	IVW	−0.436	0.230	.058	0.647	0.412	1.014
	MR Egger	−0.122	0.333	.715	0.885	0.460	1.701
	Weighted median	−0.566	0.196	.004	0.568	0.387	0.834
	Simple mode	−0.463	0.437	.294	0.629	0.267	1.482
	Weighted mode	−0.353	0.170	.043	0.703	0.503	0.981

Model 1: No confounding factors have been excluded. Model 2: Exclude TG and FPG. Model 3: Exclude TG, FPG, BMI, and metabolic symptom.

BMI = body mass index, FPG = fasting plasma glucose, IVW = inverse-variance weighted, MR = Mendelian randomization, OR = odds ratios, TG = triglycerides.

### 3.5. Subgroup analyze 3 cohort

**Figure 4.**
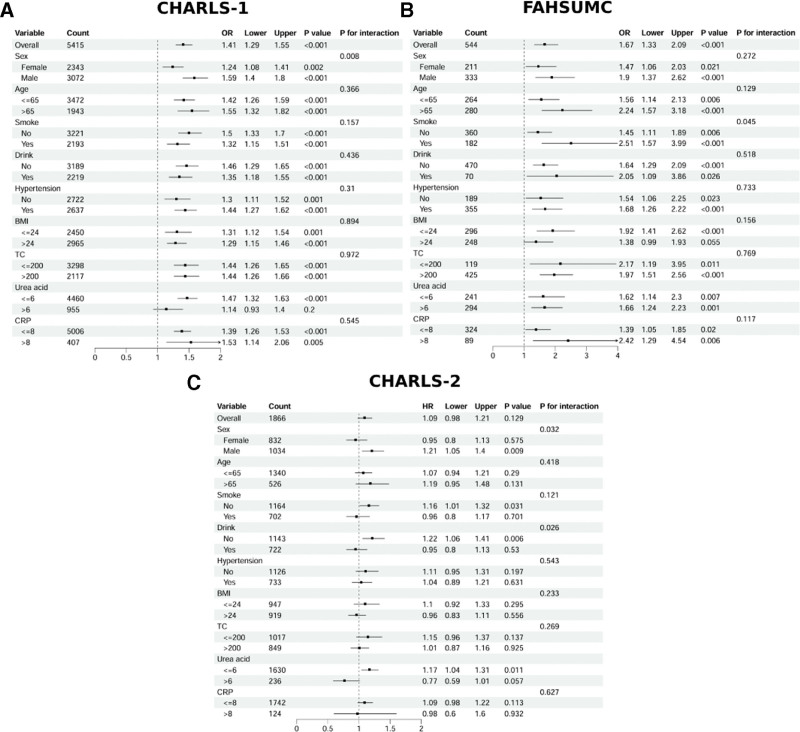
Forest plots of univariable logistic regression analysis assessing the associations between per-SD increment in TyG and CMM risk across cardiometabolic risk subgroups in the (A) CHARLS-1, (B) CHARLS-2, and (C) FAHSUMC cohorts. CHARLS = China Health and Retirement Longitudinal Study, CMM = cardiometabolic multimorbidity, FAHSUMC = First Affiliated Hospital of Shantou University Medical Center, SD = standard deviation, TyG = triglyceride-glucose.

To assess TyG-CMM associations across cardiometabolic risk strata, we conducted subgroup analyses by demographics and cardiometabolic risk factors (Fig. 4). Results aligned with primary findings: significant TyG-CMM correlations persisted across all subgroups in cross-sectional cohorts CHARLS-1 (Fig. 4A) and FAHSUMC (Fig. 4B). Conversely, in the longitudinal cohort (Fig. 4C), this association was confined to the uric acid ≤ 6 mg/dL subgroup (OR = 1.17, 95% CI: 1.04–1.31).

## 4. Discussion

This study represents the first investigation to specifically explore the relationship between the TyG index and the transition to CMM among patients with existing CMD. While our cross-sectional analyses and unadjusted longitudinal models initially suggested a predictive role for the TyG index on CMM risk, more rigorous analyses revealed critical limitations. Crucially, the apparent association lost statistical significance in the adjusted longitudinal analyses and, most definitively, in the MR analyze. This distinct pattern – where the TyG-CMM link was observed only in less controlled analyses (cross-sectional and unadjusted longitudinal) but disappeared when accounting for confounders longitudinally and under causal inference methods – provides compelling evidence that the observed association between the TyG index and CMM progression in CMD patients is likely a false-positive finding.

Recent studies have proposed the TyG index as a biomarker in the cardiometabolic era,^[24]^ with established associations between TyG and various cardiometabolic risk factors.^[25]^ Evidence further supports the link between TyG and CMM. As early as 2023, a study utilizing the CHARLS database demonstrated a strong correlation between TyG and CMM.^[13]^ Subsequent research indicated that TyG-derived insulin resistance indices (such as TyG-BMI) also correlate with CMM.^[12]^ A UK Biobank study similarly confirmed TyG’s robust association with CMM.^[26]^ However, these correlations were primarily observed in populations free of CMD at baseline. Crucially, the sole study investigating TyG’s predictive utility across preexisting CMD statuses reported that TyG lacked predictive value for CMM progression in individuals with diabetes, while its effects were marginally significant in those with CHD or stroke.^[26]^ Notably, this analysis did not adjust for key confounders such as BMI or lipid profiles, not discuss TyG as categorical variables, and the cohort differed from our target population. These limitations suggest that TyG’s role in CMM prediction warrants further investigation. Indeed, our results suggest that TyG retains a modest predictive capacity for CMM in participants with baseline CHD or stroke, even prior to comprehensive adjustment for covariates.

Given TyG’s established potency in predicting incident single CMDs^[8,10,11]^ and its documented causal relationships,^[20]^ we postulate that its primary predictive power for CMM operates predominantly through mediating transitions from 0 to 1 CMD rather than from 1 CMD to CMM.

Several limitations warrant consideration. First, our findings are primarily derived from Chinese cohorts (CHARLS and FAHSUMC), potentially limiting the generalizability to other ethnic populations with different genetic backgrounds, lifestyles, and healthcare environments. Second, while we adjusted for major known confounders (demographics, lifestyle factors, and cardiometabolic risk markers), the possibility of residual confounding by unmeasured or imprecisely measured factors (e.g., dietary habits, physical activity intensity, specific medication use, or environmental exposures) cannot be entirely excluded. Third, the longitudinal component relied on self-reported incident CMM diagnoses within CHARLS, which may introduce recall or misclassification bias despite efforts to reduce this risk through specific follow-up definitions. Fourth, the population of MR studies is from the UK, which is different from the population of observational studies. Finally, we focused solely on the TyG index; exploring interactions or combined effects with other emerging biomarkers or risk scores might provide a more comprehensive picture of CMM prediction.

## 5. Conclusions

Based on the hybrid retrospective and MR analyses, the study concludes that while cross-sectional data initially suggested an association between elevated TyG index and progression from single to multiple CMDs, adjusted longitudinal and causal inference analyses revealed no significant predictive value, indicating that earlier observed associations were likely confounded or false positives. Thus, TyG index lacks utility as a biomarker for CMM progression in established CMD patients.

## Acknowledgments

We would like to acknowledge the CHARLS research team for their dedicated time and efforts invested in the CHARLS project. We would also like to extend our gratitude to the FAHSUMC for providing access to patient data.

## Author contributions

**Data curation:** Yubo Zhang, Le Yu.

**Formal analysis:** Le Yu.

**Investigation:** Yanping Wang.

**Supervision:** Heng Wee Tan, Songming Chen.

**Validation:** Yubo Zhang.

**Writing – original draft:** Zekai Zhou, Chujuan Zeng.

**Writing – review & editing:** Zekai Zhou, Heng Wee Tan, Songming Chen.

## Supplementary Material


