# Supplementary material for: Triglyceride-glucose index demonstrates false-positive association with cardiometabolic multimorbidity progression in cardiometabolic disease patients: Observation and Mendelian randomization study
Source: Medicine (Baltimore). 2026 Feb 13;105(7):e47632. doi: 10.1097/MD.0000000000047632 (PMC12908751; doi:10.1097/MD.0000000000047632)
Supplement: Supplementary file 1 [file medi-105-e47632-s001.docx]

**Supplementary Information**

**Figure S1.** Other mendelian randomization related sensitivity analysis for model 1. MR Scatter Plot **(A)**, ​​MR Funnel Plot​​ **(B)** and ​​Leave-One-Out Sensitivity Analysis Plot​​ **(C)**.


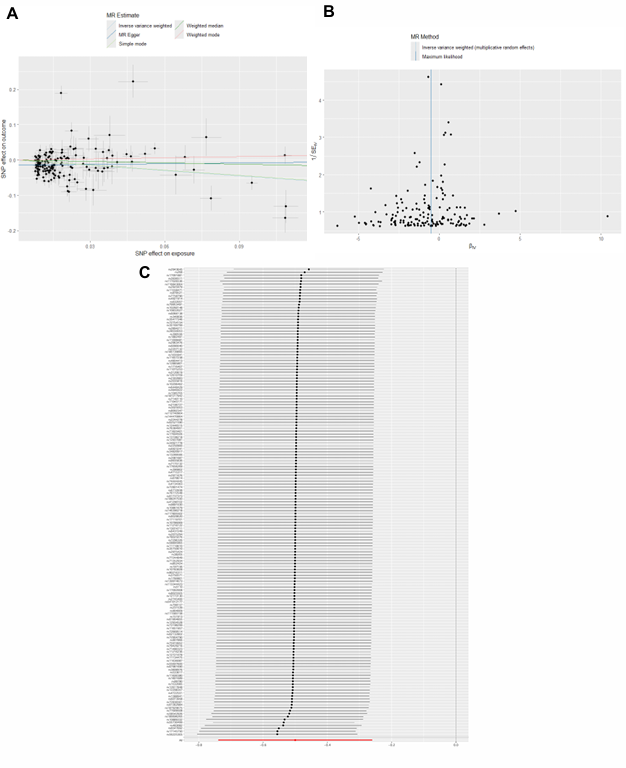


**Figure S2.** Other mendelian randomization related sensitivity analysis for model 2. MR Scatter Plot **(A)**, ​​MR Funnel Plot​​ **(B)** and ​​Leave-One-Out Sensitivity Analysis Plot​​ **(C)**.


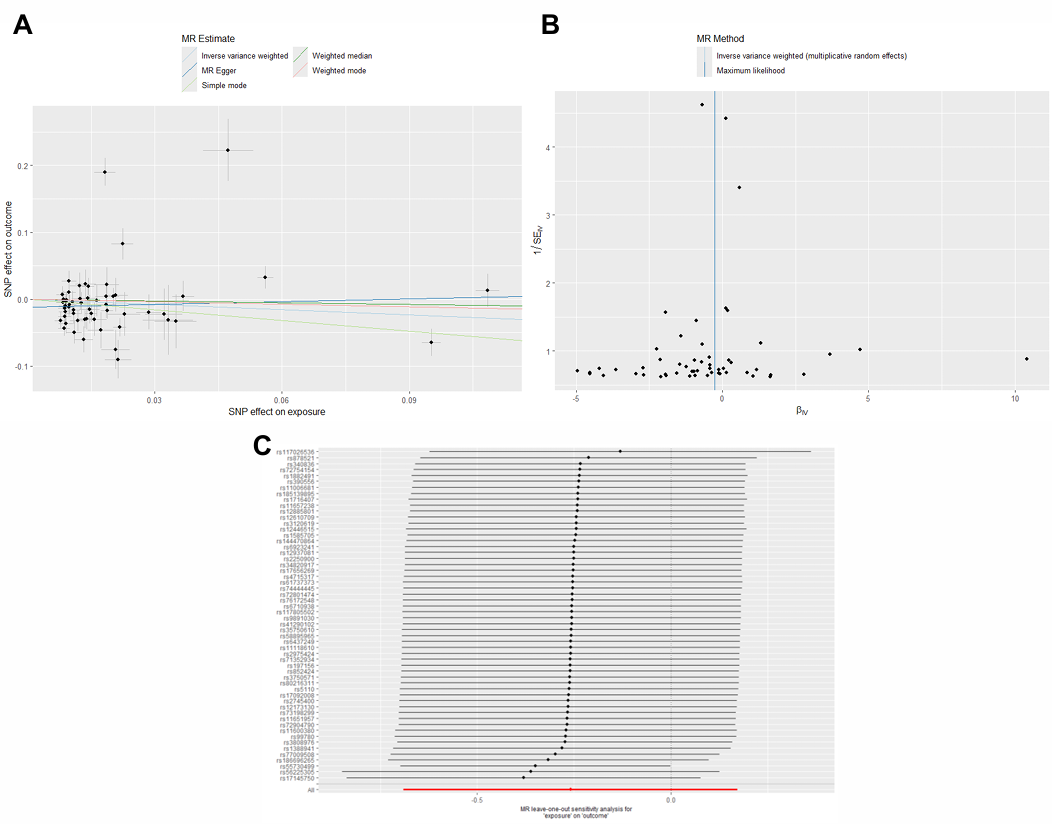


**Figure S3.** Other mendelian randomization related sensitivity analysis for model 3. MR Scatter Plot **(A)**, ​​MR Funnel Plot​​ **(B)** and ​​Leave-One-Out Sensitivity Analysis Plot​​ **(C)**.


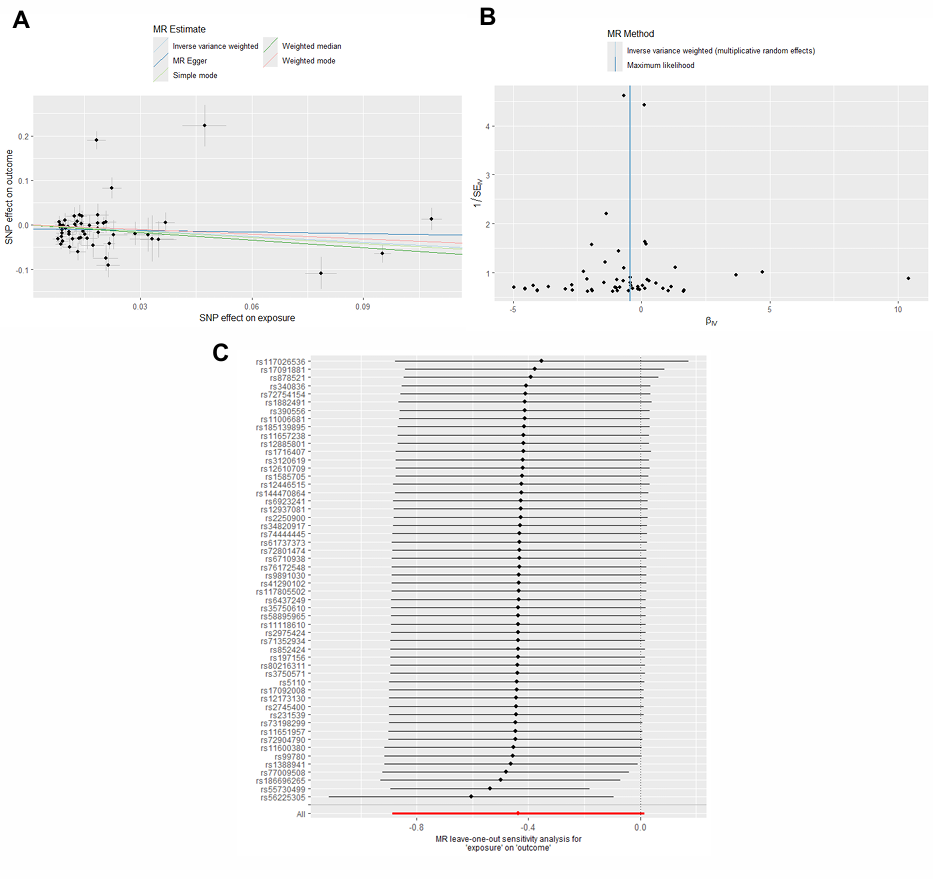


1

**Table S1.** Baseline characteristics of participants with CMD(s) (CHLARS-1 cohort)

**Table 2.** Baseline characteristics of participants with CMD(s) (FAHSUMC cohort)

**Q1**

**TyG≤8.46**

**Q2**

**Q3**

**TyG>9.13**

**Characteristics**

**Overall**

***p***

**8.46<TyG≤9.13**

**n**

**CMM (%)**

544

182

181

181

Non-CMM

CMM (%)

321 (59.0)

223 (41.0)

135 (74.2)

47 (25.8)

100 (55.2)

81 (44.8)

86 (47.5)

95 (52.5)

<0.001

<0.001

0,27

**Gender (%)**

Male

333 (61.2)

211 (38.8)

122 (67.0)

60 (33.0)

128 (70.7)

53 (29.3)

83 (45.9)

98 (54.1)

Female

**Drinke (%)**

Non-drinker

Drinker

470 (87.0)

70 (13.0)

163 (90.1)

18 ( 9.9)

156 (86.7)

24 (13.3)

151 (84.4)

28 (15.6)

**Smoke (%)**

Non-smoker

Smoker

360 (66.4)

182 (33.6)

113 (62.1)

69 (37.9)

116 (64.4)

64 (35.6)

131 (72.8)

49 (27.2)

0,078

0,046

**Hypertension (%)**

No

189 (34.7)

355 (65.3)

71 (39.0)

111 (61.0)

68 (37.6)

113 (62.4)

50 (27.6)

131 (72.4)

Yes

**Systo BP, mmHg**

**Diastolic BP, mmHg**

**Age (years)**

**Height (m)**

**Weight (kg)**

**BMI**

134.00 [122.00, 150.00]

82.00 [72.00, 90.00]

66.00 [58.00, 72.00]

1.63 (0.08)

131.00 [119.00, 147.00]

80.00 [71.25, 89.00]

68.00 [60.25, 74.00]

1.63 (0.08)

131.00 [116.00, 148.00]

79.50 [71.00, 90.25]

65.00 [57.00, 70.00]

1.65 (0.08)

139.00 [126.00, 156.00]

85.00 [76.00, 93.00]

65.00 [57.00, 71.00]

1.62 (0.08)

0,001

0,008

0,002

0,008

63.80 (11.00)

62.25 (11.70)

65.19 (10.28)

63.96 (10.82)

0,038

23.64 [21.61, 25.89]

103.68 [86.58, 144.18]

257.11 [210.50, 319.32]

116.95 [80.63, 167.45]

61.20 [50.91, 72.07]

100.23 [69.95, 132.16]

2.30 [0.90, 6.40]

22.88 [20.76, 25.22]

85.50 [77.81, 93.56]

233.38 [185.61, 273.42]

70.44 [59.36, 86.61]

66.64 [53.91, 78.79]

90.95 [59.31, 116.78]

2.35 [0.70, 9.15]

23.88 [22.21, 26.03]

105.84 [90.90, 123.84]

263.69 [212.21, 323.75]

120.50 [101.89, 143.53]

60.63 [53.20, 70.36]

109.91 [73.14, 143.58]

2.00 [0.90, 5.77]

24.03 [22.04, 26.40]

159.12 [119.52, 213.66]

288.86 [233.38, 363.79]

202.89 [147.96, 284.41]

57.77 [45.76, 68.07]

102.56 [73.92, 142.80]

2.40 [1.10, 6.70]

0,001

**Glucose (mg/dl)**

**TC (mmol/L)**

**TG (mmol/L)**

**HDL-C (mmol/L)**

**LDL-C (mmol/L)**

**CRP (mg/L)**

**HbA1c**

<0.001

<0.001

<0.001

<0.001

<0.001

0,338

6.86 [6.12, 8.63]

6.13 [5.82, 6.72]

6.80 [6.22, 7.90]

8.61 [7.06, 10.86]

6.40 [5.27, 7.51]

<0.001

0,005

**Uric Acid**

6.17 [5.11, 7.39]

5.90 [4.81, 7.08]

6.46 [5.38, 7.54]

**TyG**

8.79 [8.28, 9.36]

8.04 [7.85, 8.28]

8.79 [8.63, 8.94]

9.58 [9.36, 9.97]

<0.001

1

**Table S3.** Baseline characteristics of participants with CMD(s) (CHLARS-2 cohort)

**Table S4.** Missing values of the CHARLS-1 and CHARLS-2 populations

|  | **CHLARS 1** | | **CHLARS 2** | |
| --- | --- | --- | --- | --- |
| **Variable** | **Missing (n)** | **Missing (%)** | **Missing (n)** | **Missing (%)** |
| Gender | 0 | 0 | 0 | 0 |
| Drinke | 7 | 0.13 | 1 | 0.05 |
| Smoke | 1 | 0.02 | 0 | 0 |
| Exercise | 24 | 0.44 | 15 | 0.80 |
| Hospitalized in the past year | 8 | 0.15 | 0 | 0 |
| Hypertension | 56 | 1.03 | 7 | 0.38 |
| Systolic | 73 | 1.35 | 19 | 1.02 |
| Diastolic | 74 | 1.37 | 19 | 1.02 |
| Income | 2356 | 43.51 | 627 | 33.60 |
| Age | 0 | 0 | 0 | 0 |
| Waistline | 9 | 0.17 | 0 | 0 |
| Sleeptime | 149 | 2.75 | 29 | 1.55 |
| TC | 0 | 0 | 0 | 0 |
| HDL-C | 0 | 0 | 0 | 0 |
| LDL-C | 15 | 0.28 | 12 | 0.64 |
| CRP | 2 | 0.04 | 0 | 0 |
| HbA1c | 14 | 0.26 | 8 | 0.43 |
| Uric Acid | 0 | 0 | 0 | 0 |

**Table S5.** Missing values of the FAHSUMC populations

| **Variable** | **Missing (n)** | **Missing (%)** |
| --- | --- | --- |
| Gender | 0 | 0 |
| Drinke | 4 | 0.74 |
| Smoke | 3 | 0.36 |
| Hypertension | 0 | 0 |
| Systolic | 3 | 0.55 |
| Diastolic | 3 | 0.55 |
| Age | 0 | 0 |
| TC | 0 | 0 |
| HDL-C | 0 | 0 |
| LDL-C | 0 | 0 |
| CRP | 131 | 24.08 |
| HbA1c | 55 | 10.11 |
| Uric Acid | 9 | 1.65 |

**Table S6.** CHARLS-1 Kolmogorov-Smirnov and CHARLS-2 Shapiro-Wilk test results

|  | | **CHLARS-1** | | |  | **CHLARS-2** | | |
| --- | --- | --- | --- | --- | --- | --- | --- | --- |
| **Characteristic** | **D** | | **P value** |  |  | **W** | **P value** |  |
| TyG | 0.05652 | | 1.888E-15 | nonnormal |  | 0.95546 | < 2.2e-16 | nonnormal |
| Uric Acid | 0.97751 | | < 2.2e-16 | nonnormal |  | 0.97147 | < 2.2e-16 | nonnormal |
| HBA1C | 0.99977 | | < 2.2e-16 | nonnormal |  | 0.73508 | < 2.2e-16 | nonnormal |
| CRP | 0.58744 | | < 2.2e-16 | nonnormal |  | 0.32843 | < 2.2e-16 | nonnormal |
| LDL | 0.99926 | | < 2.2e-16 | nonnormal |  | 0.98517 | 6.316e-13 | nonnormal |
| HDL | 1 | | < 2.2e-16 | nonnormal |  | 0.95486 | < 2.2e-16 | nonnormal |
| TG | 0.99982 | | < 2.2e-16 | nonnormal |  | 0.56641 | < 2.2e-16 | nonnormal |
| TC | 1 | | < 2.2e-16 | nonnormal |  | 0.94112 | < 2.2e-16 | nonnormal |
| FG | 0.84134 | | < 2.2e-16 | nonnormal |  | 0.73743 | < 2.2e-16 | nonnormal |
| BMI | 0.99982 | | < 2.2e-16 | nonnormal |  | 0.17749 | < 2.2e-16 | nonnormal |
| Waist | 1 | | < 2.2e-16 | nonnormal |  | 0.90908 | < 2.2e-16 | nonnormal |
| Weight | 0.99982 | | < 2.2e-16 | nonnormal |  | 0.96215 | < 2.2e-16 | nonnormal |
| Height | 0.90804 | | < 2.2e-16 | nonnormal |  | 0.92441 | < 2.2e-16 | nonnormal |
| DBP | 1 | | < 2.2e-16 | nonnormal |  | 0.99126 | 4.491e-09 | nonnormal |
| SBP | 1 | | < 2.2e-16 | nonnormal |  | 0.97076 | < 2.2e-16 | nonnormal |
| Age | 1 | | < 2.2e-16 | nonnormal |  | 0.97789 | < 2.2e-16 | nonnormal |
| Income | 0.92285 | | < 2.2e-16 | nonnormal |  | 0.74049 | < 2.2e-16 | nonnormal |

**Table S7.** FAHSUMC Shapiro-Wilk test results

| **Characteristic** | **W** | **P value** |  |
| --- | --- | --- | --- |
| TyG | 0.97405 | 3.122E-08 | nonnormal |
| TyG-BMI | 0.98551 | 3.086E-05 | nonnormal |
| Uric Acid | 0.99102 | 0.002461 | nonnormal |
| HBA1C | 0.84021 | < 2.2e-16 | nonnormal |
| CRP | 0.40463 | < 2.2e-16 | nonnormal |
| LDL | 0.97947 | 6.275E-07 | nonnormal |
| HDL | 0.95447 | 6.526E-12 | nonnormal |
| TG | 0.64298 | < 2.2e-16 | nonnormal |
| TC | 0.97875 | 4.12E-07 | nonnormal |
| FG | 0.80969 | < 2.2e-16 | nonnormal |
| BMI | 0.99035 | 0.00124 | nonnormal |
| Weight | 0.97964 | 6.962E-07 | nonnormal |
| Height | 0.98815 | 0.0002159 | nonnormal |
| diasto | 0.99024 | 0.001186 | nonnormal |
| systo | 0.98248 | 4.285E-06 | nonnormal |
| Age | 0.99122 | 0.00259 | nonnormal |

**Table S8.** TyG related SNP data

| **SNP** | **REF** | **ALT** | **BETA** | **SE** | **P** |
| --- | --- | --- | --- | --- | --- |
| rs114165349 | G | C | 0.04453 | 0.004653 | 1.07E-21 |
| rs72904790 | T | C | 0.013715 | 0.002406 | 1.2E-08 |
| rs213498 | T | A | -0.00803 | 0.001464 | 4.17E-08 |
| rs10889332 | C | T | -0.0389 | 0.00143 | 1.3E-162 |
| rs72669514 | C | T | 0.018609 | 0.003207 | 6.53E-09 |
| rs17656269 | C | T | 0.008749 | 0.00147 | 2.65E-09 |
| rs16836630 | G | C | -0.01734 | 0.002521 | 6.14E-12 |
| rs1760801 | G | A | -0.00896 | 0.001513 | 3.19E-09 |
| rs340836 | T | C | -0.0087 | 0.001396 | 4.71E-10 |
| rs76172548 | A | C | 0.022814 | 0.003832 | 2.64E-09 |
| rs3120619 | G | A | 0.011881 | 0.001803 | 4.45E-11 |
| rs11118610 | A | C | -0.00903 | 0.001389 | 8.09E-11 |
| rs4846922 | C | T | 0.022155 | 0.001463 | 8.63E-52 |
| rs907866 | G | A | -0.00928 | 0.001388 | 2.31E-11 |
| rs111585158 | C | T | 0.012113 | 0.002114 | 1E-08 |
| rs144470864 | A | C | 0.017261 | 0.003078 | 2.05E-08 |
| rs76384951 | A | C | -0.02955 | 0.002524 | 1.16E-31 |
| rs533617 | T | C | -0.04645 | 0.003467 | 6.48E-41 |
| rs35750610 | T | C | 0.018513 | 0.002347 | 3.09E-15 |
| rs34921778 | A | G | 0.008426 | 0.00145 | 6.21E-09 |
| rs12617848 | C | T | 0.014279 | 0.002006 | 1.09E-12 |
| rs80216311 | C | T | -0.01417 | 0.00239 | 3.05E-09 |
| rs61737373 | G | A | -0.02861 | 0.002929 | 1.57E-22 |
| rs6547692 | A | G | 0.037507 | 0.001384 | 2.1E-161 |
| rs10206462 | T | C | -0.00894 | 0.001428 | 3.85E-10 |
| rs6760053 | C | G | -0.00779 | 0.001377 | 1.55E-08 |
| rs6710938 | A | C | -0.00899 | 0.001618 | 2.76E-08 |
| rs79953491 | A | G | -0.0237 | 0.002108 | 2.49E-29 |
| rs115128825 | C | A | 0.026901 | 0.004876 | 3.46E-08 |
| rs484066 | T | A | -0.01592 | 0.001419 | 3.31E-29 |
| rs17694506 | T | C | 0.009005 | 0.001419 | 2.19E-10 |
| rs2943645 | T | C | -0.02092 | 0.001432 | 2.4E-48 |
| rs6437249 | C | T | 0.008385 | 0.001493 | 1.95E-08 |
| rs147764624 | G | C | -0.03019 | 0.005506 | 4.18E-08 |
| rs390802 | G | A | -0.01534 | 0.001765 | 3.63E-18 |
| rs62271373 | T | A | 0.025359 | 0.003039 | 7.15E-17 |
| rs13074711 | T | C | 0.012011 | 0.002185 | 3.86E-08 |
| rs13108218 | G | A | 0.01564 | 0.001438 | 1.54E-27 |
| rs71603401 | A | G | 0.012509 | 0.002055 | 1.15E-09 |
| rs6448429 | C | T | 0.012675 | 0.001878 | 1.49E-11 |
| rs1471251 | A | T | 0.016447 | 0.001408 | 1.56E-31 |
| rs4134363 | G | A | -0.0095 | 0.001702 | 2.37E-08 |
| rs3822076 | T | A | 0.008671 | 0.001382 | 3.5E-10 |
| rs2035816 | A | G | -0.01594 | 0.002491 | 1.54E-10 |
| rs78025076 | C | T | 0.027112 | 0.004828 | 1.96E-08 |
| rs390556 | T | C | -0.01327 | 0.002204 | 1.73E-09 |
| rs72754154 | G | A | -0.0214 | 0.003141 | 9.58E-12 |
| rs3936511 | A | G | 0.021613 | 0.001747 | 3.69E-35 |
| rs151913 | G | A | 0.00787 | 0.001414 | 2.6E-08 |
| rs7703744 | C | G | -0.01093 | 0.001552 | 1.84E-12 |
| rs72801474 | G | A | -0.01464 | 0.002353 | 4.88E-10 |
| rs12173130 | T | C | 0.009711 | 0.001769 | 4.07E-08 |
| rs11134475 | G | A | -0.01694 | 0.001423 | 1.15E-32 |
| rs2963476 | A | G | 0.013369 | 0.001699 | 3.58E-15 |
| rs6923241 | C | T | -0.01092 | 0.001547 | 1.67E-12 |
| rs2745400 | G | A | 0.00826 | 0.001372 | 1.76E-09 |
| rs2894211 | C | A | 0.017465 | 0.002189 | 1.46E-15 |
| rs7758790 | T | C | 0.01428 | 0.001648 | 4.52E-18 |
| rs55697600 | A | G | 0.035107 | 0.003604 | 2.05E-22 |
| rs185139895 | G | A | 0.020756 | 0.003384 | 8.63E-10 |
| rs3025053 | G | A | -0.01346 | 0.002125 | 2.4E-10 |
| rs4715317 | G | T | 0.009743 | 0.001439 | 1.3E-11 |
| rs1967685 | G | C | -0.01428 | 0.001371 | 2.25E-25 |
| rs632057 | G | T | 0.015367 | 0.001421 | 2.94E-27 |
| rs12208357 | C | T | 0.021956 | 0.002725 | 7.75E-16 |
| rs77009508 | A | G | 0.022418 | 0.002596 | 5.86E-18 |
| rs55730499 | C | T | -0.01825 | 0.002552 | 8.74E-13 |
| rs186696265 | C | T | -0.04726 | 0.005925 | 1.51E-15 |
| rs4709746 | C | T | -0.01125 | 0.002032 | 3.11E-08 |
| rs852424 | C | T | 0.008528 | 0.001463 | 5.55E-09 |
| rs38205 | C | A | 0.007915 | 0.00144 | 3.87E-08 |
| rs2106727 | G | A | -0.01082 | 0.001428 | 3.59E-14 |
| rs4722551 | T | C | -0.01858 | 0.001881 | 5.21E-23 |
| rs1534696 | A | C | 0.010737 | 0.001376 | 6.18E-15 |
| rs2971676 | G | A | 0.013349 | 0.002398 | 2.61E-08 |
| rs878521 | G | A | 0.021762 | 0.001587 | 8.43E-43 |
| rs62459110 | G | C | -0.02139 | 0.003643 | 4.32E-09 |
| rs799157 | C | T | 0.040791 | 0.003402 | 4.05E-33 |
| rs17145750 | C | T | -0.05606 | 0.001856 | 5.3E-200 |
| rs10260148 | C | T | 0.014959 | 0.001542 | 2.96E-22 |
| rs73198299 | T | C | 0.012271 | 0.002229 | 3.68E-08 |
| rs7821812 | G | C | 0.016336 | 0.001697 | 6.39E-22 |
| rs904009 | A | C | 0.015931 | 0.001626 | 1.16E-22 |
| rs4921914 | T | C | 0.019439 | 0.001659 | 1.07E-31 |
| rs2975424 | T | C | 0.010605 | 0.001755 | 1.53E-09 |
| rs1388941 | G | A | 0.014354 | 0.001459 | 8.01E-23 |
| rs268 | A | G | 0.10865 | 0.005152 | 1.25E-98 |
| rs117026536 | G | T | -0.09518 | 0.002263 | 0 |
| rs57295072 | G | C | -0.03049 | 0.004697 | 8.6E-11 |
| rs17091881 | T | C | 0.078595 | 0.004274 | 1.82E-75 |
| rs74444445 | T | C | 0.034928 | 0.004886 | 8.82E-13 |
| rs117805502 | C | T | -0.03217 | 0.004394 | 2.48E-13 |
| rs28550053 | A | G | -0.01771 | 0.001821 | 2.42E-22 |
| rs75662196 | G | C | -0.02793 | 0.004345 | 1.3E-10 |
| rs17092008 | C | T | 0.020825 | 0.002853 | 2.91E-13 |
| rs11781356 | T | A | 0.009932 | 0.001766 | 1.85E-08 |
| rs2081687 | C | T | 0.011677 | 0.001454 | 9.63E-16 |
| rs71525127 | C | G | 0.019603 | 0.002547 | 1.42E-14 |
| rs11558471 | A | G | -0.01148 | 0.001471 | 6.29E-15 |
| rs17321515 | A | G | -0.0439 | 0.001371 | 2E-224 |
| rs62521590 | T | G | 0.014654 | 0.001558 | 5.11E-21 |
| rs10811661 | T | C | -0.00987 | 0.001805 | 4.53E-08 |
| rs13289566 | C | T | -0.01183 | 0.001669 | 1.37E-12 |
| rs2244278 | C | A | -0.01339 | 0.002119 | 2.62E-10 |
| rs3750571 | C | A | -0.01241 | 0.001898 | 6.27E-11 |
| rs11006681 | G | A | -0.01101 | 0.001844 | 2.38E-09 |
| rs142164605 | T | A | -0.01776 | 0.002781 | 1.71E-10 |
| rs10786069 | T | C | 0.013098 | 0.001378 | 2.06E-21 |
| rs113344423 | G | A | 0.021299 | 0.003018 | 1.7E-12 |
| rs2792736 | A | T | -0.01005 | 0.001538 | 6.4E-11 |
| rs10832027 | A | G | -0.01226 | 0.001482 | 1.37E-16 |
| rs3808976 | A | G | 0.009819 | 0.001701 | 7.84E-09 |
| rs99780 | C | T | 0.020203 | 0.001436 | 5.73E-45 |
| rs35169799 | C | T | 0.024724 | 0.002833 | 2.61E-18 |
| rs678614 | C | A | 0.009351 | 0.001532 | 1.04E-09 |
| rs2302883 | T | C | 0.008865 | 0.001623 | 4.71E-08 |
| rs187217942 | G | A | 0.031159 | 0.005403 | 8.06E-09 |
| rs17119701 | A | G | 0.037068 | 0.00375 | 4.94E-23 |
| rs61362984 | A | G | -0.01395 | 0.001422 | 1.06E-22 |
| rs61904855 | C | A | 0.023378 | 0.004096 | 1.15E-08 |
| rs11216122 | G | T | -0.01819 | 0.003103 | 4.59E-09 |
| rs7930786 | G | C | 0.124688 | 0.002787 | 0 |
| rs56225305 | G | A | 0.108415 | 0.002786 | 0 |
| rs2075294 | G | T | 0.038812 | 0.005799 | 2.19E-11 |
| rs75919952 | C | T | -0.04681 | 0.00317 | 2.56E-49 |
| rs11600380 | T | C | -0.03673 | 0.002541 | 2.34E-47 |
| rs5110 | C | A | -0.01854 | 0.002485 | 8.59E-14 |
| rs12721078 | C | A | -0.03228 | 0.003938 | 2.46E-16 |
| rs71480323 | G | A | -0.01956 | 0.002114 | 2.24E-20 |
| rs11216236 | C | T | 0.024039 | 0.003424 | 2.21E-12 |
| rs187929675 | C | T | -0.07679 | 0.006068 | 1.07E-36 |
| rs11045171 | A | G | -0.01166 | 0.001737 | 1.91E-11 |
| rs67981690 | A | G | 0.014856 | 0.002075 | 8.09E-13 |
| rs10783828 | G | A | 0.009035 | 0.001475 | 9.04E-10 |
| rs7296326 | T | C | -0.01186 | 0.002173 | 4.84E-08 |
| rs1585705 | A | C | 0.008766 | 0.001496 | 4.64E-09 |
| rs10861679 | T | C | 0.009354 | 0.001507 | 5.35E-10 |
| rs1882491 | T | C | -0.0135 | 0.001481 | 8.04E-20 |
| rs1716407 | A | G | -0.01506 | 0.001399 | 4.9E-27 |
| rs7140110 | T | C | 0.01437 | 0.001508 | 1.61E-21 |
| rs112740904 | T | G | -0.0149 | 0.001958 | 2.76E-14 |
| rs12885801 | C | A | 0.009085 | 0.001622 | 2.11E-08 |
| rs34820917 | G | A | -0.0158 | 0.00285 | 2.97E-08 |
| rs35477346 | T | C | 0.009297 | 0.001497 | 5.32E-10 |
| rs139974673 | T | C | 0.071769 | 0.00443 | 5.34E-59 |
| rs72739147 | A | T | -0.01212 | 0.002061 | 4.04E-09 |
| rs1532085 | G | A | 0.018004 | 0.00141 | 2.64E-37 |
| rs261334 | C | G | 0.026145 | 0.001678 | 9.88E-55 |
| rs11636087 | T | C | 0.011653 | 0.001545 | 4.65E-14 |
| rs8028620 | T | C | -0.00898 | 0.001375 | 6.53E-11 |
| rs7175132 | A | G | -0.00812 | 0.001414 | 9.44E-09 |
| rs8025505 | C | T | 0.009647 | 0.001584 | 1.13E-09 |
| rs9935836 | A | C | 0.009882 | 0.001773 | 2.49E-08 |
| rs11075253 | C | A | -0.01411 | 0.001502 | 5.65E-21 |
| rs12446515 | C | T | -0.01876 | 0.001475 | 4.76E-37 |
| rs5880 | G | C | 0.022114 | 0.002981 | 1.18E-13 |
| rs12934528 | T | C | 0.013515 | 0.001958 | 5.15E-12 |
| rs2925979 | C | T | 0.015438 | 0.001498 | 6.79E-25 |
| rs11651957 | G | A | 0.018648 | 0.002936 | 2.14E-10 |
| rs12937081 | A | G | 0.010884 | 0.001891 | 8.64E-09 |
| rs72836561 | C | T | 0.068218 | 0.003899 | 1.69E-68 |
| rs231539 | C | T | 0.013065 | 0.001881 | 3.76E-12 |
| rs11657238 | G | A | -0.00785 | 0.001384 | 1.41E-08 |
| rs1801689 | A | C | -0.02932 | 0.004081 | 6.78E-13 |
| rs77244849 | T | C | -0.00875 | 0.001488 | 4.09E-09 |
| rs9891030 | G | A | 0.009938 | 0.001594 | 4.54E-10 |
| rs71352934 | A | C | -0.01631 | 0.002736 | 2.5E-09 |
| rs8092347 | A | G | 0.008121 | 0.001411 | 8.69E-09 |
| rs197156 | A | G | -0.00925 | 0.001454 | 1.99E-10 |
| rs1035941 | G | A | 0.011015 | 0.001533 | 6.75E-13 |
| rs4804413 | C | T | 0.009485 | 0.001385 | 7.53E-12 |
| rs116843064 | G | A | -0.10888 | 0.004912 | 8.8E-109 |
| rs57192995 | G | A | -0.0199 | 0.003024 | 4.65E-11 |
| rs58542926 | C | T | -0.05205 | 0.002577 | 1.26E-90 |
| rs188247550 | C | T | -0.06447 | 0.006421 | 1.02E-23 |
| rs62102718 | A | T | 0.011589 | 0.001527 | 3.22E-14 |
| rs58895965 | C | A | 0.012724 | 0.001804 | 1.75E-12 |
| rs541012177 | G | T | 0.024254 | 0.003536 | 6.94E-12 |
| rs41290102 | C | T | -0.03312 | 0.005888 | 1.86E-08 |
| rs419925 | G | C | -0.01304 | 0.001499 | 3.39E-18 |
| rs483082 | G | T | 0.044675 | 0.001617 | 8.1E-168 |
| rs79429216 | G | A | 0.037821 | 0.006299 | 1.92E-09 |
| rs146390218 | A | G | 0.035524 | 0.004351 | 3.22E-16 |
| rs62132802 | C | T | -0.00912 | 0.001502 | 1.29E-09 |
| rs12610709 | G | A | 0.013994 | 0.001833 | 2.24E-14 |
| rs2207132 | G | A | 0.028323 | 0.003878 | 2.83E-13 |
| rs2250900 | C | T | 0.008996 | 0.00164 | 4.1E-08 |
| rs6073958 | T | C | 0.027409 | 0.001724 | 7.14E-57 |
| rs4812995 | T | C | 0.009133 | 0.001617 | 1.64E-08 |
| rs6066138 | G | A | -0.00851 | 0.001523 | 2.36E-08 |
| rs6090040 | C | A | 0.008917 | 0.001388 | 1.33E-10 |
| rs2277844 | A | G | -0.00908 | 0.001385 | 5.43E-11 |

**Table S9.** Mendelian randomization Cochran’s Q test and MR-Egger intercept test

|  | **Heterogeneity** | | | **Horizontal pleiotropy** | | |
| --- | --- | --- | --- | --- | --- | --- |
|  | **Q** | **Q df** | **P value** | **Egger intercept** | **SE** | **P value** |
| Model 1 | 510.4264 | 157 | <0.001 | 0.01438 | 0.003795 | <0.001 |
| Model 2 | 243.517 | 55.000 | <0.001 | -0.012 | 0.007 | 0.091 |
| Model 3 | 234.024 | 53 | <0.001 | -0.009 | 0.007 | 0.202 |
